# A New and Practical Synthetic Method for the Synthesis of 6-*O*-Methyl-scutellarein: One Metabolite of Scutellarin *in Vivo*

**DOI:** 10.3390/ijms16047587

**Published:** 2015-04-03

**Authors:** Hang Lin, Wei Zhang, Ze-Xi Dong, Ting Gu, Nian-Guang Li, Zhi-Hao Shi, Jun Kai, Cheng Qu, Guan-Xiong Shang, Yu-Ping Tang, Fang Fang, He-Min Li, Jian-Ping Yang, Jin-Ao Duan

**Affiliations:** 1 National and Local Collaborative Engineering Center of Chinese Medicinal Resources Industrialization and Formulae Innovative Medicine, Nanjing University of Chinese Medicine, Nanjing 210023, Jiangsu, China; E-Mails: lhcywww@126.com (H.L.); zhangwei20131405@163.com (W.Z.); dongzexi1215@163.com (Z.-X.D.); guting1992@163.com (T.G.); kaijun2015@126.com (J.K.); qucheng9527@163.com (C.Q.); guanxiongshang2015@126.com (G.-X.S.); fylffh@163.com (F.F.); lihemin2006@sina.com (H.-M.L.); jpyang-0828@163.com (J.-P.Y.); dja@njutcm.edu.cn (J.-A.D.); 2 Jiangsu Key Laboratory for High Technology Research of TCM Formulae, Nanjing University of Chinese Medicine, Nanjing 210023, Jiangsu, China; 3 Jiangsu Collaborative Innovation Center of Chinese Medicinal Resources Industrialization, Nanjing University of Chinese Medicine, Nanjing 210023, Jiangsu, China; 4 Department of Organic Chemistry, China Pharmaceutical University, Nanjing 211198, Jiangsu, China; E-Mail: sszh163@163.com

**Keywords:** 6-*O*-methyl-scutellarein, scutellarin, scutellarein, metabolite, synthesis

## Abstract

Scutellarin (**1**) has been used for the treatment of angina pectoris, cerebral infarction and coronary heart disease with a large market share in China. Pharmacokinetic studies on scutellarin showed that scutellarin (**1**) is readily converted into its metabolites *in vivo*. In this paper, a new and practical synthetic method for the synthesis of 6-*O*-methyl-scutellarein (**3**) (one metabolite of scutellarin *in vivo*) is reported. The benzyl bromide was firstly used to selectively replace the acetyl group at C-7 in **7**, and was then used to protect the hydroxy groups at C-4' in **10**, 6-*O*-methyl-scutellarein (**3**) is obtained in high yield through these methods.

## 1. Introduction

**Figure 1:**
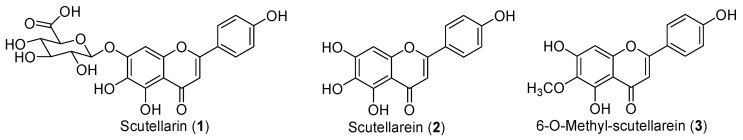
Chemical structures of scutellarin (**1**), scutellarein (**2**) and 6-*O*-methyl-scutellarein (**3**).

Ischemic cerebrovascular disease is a frequently-occurring disease, and it has been one of the leading causes of death and disability worldwide that seriously endangers human health [1]. Traditional Chinese medicines have been used clinically for thousands years and can be regarded as potential rich sources for drug lead compound discovery. Scutellarin (**1**) (Figure 1), which is the main effective constituent (>85%) of breviscapine, a natural drug consisting of total flavonoids of Erigeron breviscapus (Vant.) Hand-Mazz. (Compositae), has been used for the treatment of angina pectoris, cerebral infarction, and coronary heart disease and has a large market share in China [2]. Nowadays, the research on scutellarin (**1**) has become a hot topic in China due to its distinguished efficacy in the clinical therapy. Pharmacological studies found that scutellarin (**1**) demonstrated antioxidant and anticoagulant activities to attenuate neuronal damage, thus had a wide range of benefits to brain injury caused by cerebral ischemia/reperfusion [3,4,5].

Recently, pharmacokinetic studies on scutellarin (**1**) showed that the oral bioavailability of scutellarin (**1**) was quite poor [6] in rats [7,8,9], dogs [10], and humans [11]. One reason was its poor aqueous solubility and low lipophilicity [12], and its poor ability to penetrate cell membranes has hindered its overall effectiveness as an oral drug. The other reason was that scutellarin (**1**) is readily hydrolyzed into scutellarein (**2**) (Figure 1) before absorption *in vivo*. Scutellarein (**2**) is relatively easily absorbed into the blood and can metabolite into methylated, sulfated or glucuronidated forms [13]. 6-*O*-methyl-scutellarein (**3**) (Figure 1), which is one of the circulating metabolite of scutellarin *in vivo*, might be responsible for the therapeutic effects of scutellarin (**1**). However, this interesting scutellarin metabolite (**3**) is not commercially available, as a result, synthetic methods for this metabolite will be very important for further study.

**Figure 2:**
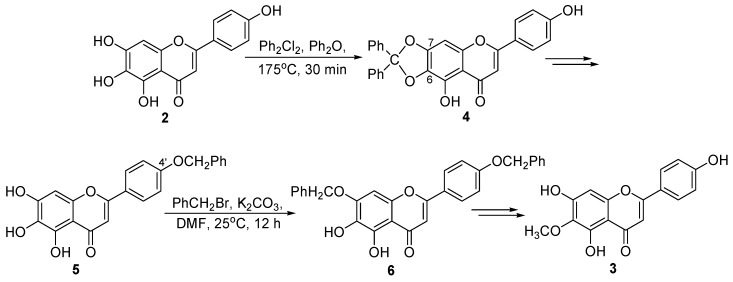
The previous synthetic route of 6-*O*-methyl-scutellarein (**3**).

With an aim to provide a solution to the material supply issue we recently reported a synthetic route to 6-*O*-methyl-scutellarein (**3**) (Figure 2) [14]. This method firstly used dichlorodiphenylmethane to protect the dihydroxy groups at C-6 and C-7 in scutellarein (**4**). Subsequently, benzyl bromide was used to selectively protect the hydroxy groups at C-4' in 5 and at C-7 in **6**, respectively. Finally, 6-*O*-methyl-scutellarein (**3**) was obtained after methylation at C-6 followed by hydrogenation of the two benzyl groups at C-7 and C-4'.

However, the reaction condition using dichlorodiphenylmethane at 175 °C [14] to protect the dihydroxy groups at C-6 and C-7 in scutellarein (**2**) would hamper the large-scale preparation of 6-*O*-methyl-scutellarein (**3**). After an extensive study, we have exploited a new and practical synthesis of 6-*O*-methyl-scutellarein (**3**) by first using acetic anhydride as the protecting group instead. Herein, we report this synthetic route in detail.

## 2. Results and Discussion

**Scheme 1:**
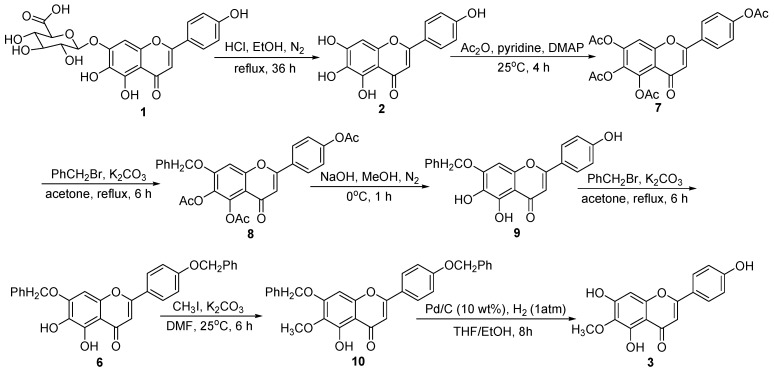
Synthetic route of 6-*O*-methyl-scutellarein (**3**).

As shown in Scheme 1, 6-*O*-methyl-scutellarein (**3**) was synthesized from scutellarin (**1**) in seven steps. According to our previous method [15,16], the scutellarein (**2**) was obtained from the scutellarin (**1**) by refluxing it with 6 N HCl in 90% ethanol under the N_2_ protection. Then acetic anhydride was used to protect all the hydroxyl groups in **2** to give compound **7** in 90% yields. Interestingly, the reaction of **7** with benzyl bromide issued the substitution of acetic group by benzyl group to afford **8**, and this chemical structure was confirmed by 1H NMR and ROESY. In the 1H NMR spectrum (in DMSO-*d*_6_) of **8**, the presence of three single signals at 2.31, 2.32, and 2.34, respectively, indicated that the three acetic groups remained unchanged, one single signal at 5.36 and multi signals at 7.36–7.45 showed that there is one benzyl group in compound **8**. The benzyl group position in **8** was confirmed by ROESY spectrum, and a cross-peak between 5.36 (–OCH_2_) with 6.87 (C8–H) indicated that the benzyl group was at C7–OH position. This preferential reaction [17,18] might be because the electronically deficient C-4 carbonyl group of **7** was para to the C-7 acetoxy unit. Thus, C-4 carbonyl group could accept electron density from the C-7 oxygen, which contributed to a weakening of the ester bond and facilitated the rupture of the bond, leading to the benzyl substituted intermediate **8**.

Deprotection of the acetyl groups in **8** with NaOH (aq) afforded **9** in 90% yield, and we then turned our attention to selectively protect the hydroxyl group at C-4' in **9**. There were three hydroxyl groups in **9**, however, their reactive activities were different and they were in the preferential order 4' > 6 > 5 [19]. Thus, the treatment of **9** with 1.5 equivalent of benzyl bromide in the presence of K_2_CO_3_ led to the di-benzyl ether substituted product **6**. A cross-peak observed in the ROESY spectrum of **6** between 5.03 (–OCH_2_Ph) with 6.87 (C8–H) indicated that one of the two benzyl group was at C7–OH, other cross-peak between 5.23 (–OCH_2_Ph) with 7.18 (C3',5'-H) indicated that the other benzyl group was at C4'–OH. Treatment of **6** with 1.2 equiv. of iodomethane led selectively to **10** with the desired methyl group in C6–OH in 94% yield. Then, the deprotection of di-benzyl groups under hydrogenation conditions using 10% palladium on carbon as the catalyst in THF/EtOH afforded **3** in 96% yield.

## 3. Experimental Section 

Reagents and solvents were purchased from commercial sources and used without further purification unless otherwise specified. Air- and moisture-sensitive liquids and solutions were transferred via syringe or stainless steel cannula. Organic solutions were concentrated by rotary evaporation below 45 °C at approximately 20 mm Hg. All non-aqueous reactions were carried out under anhydrous conditions using flame-dried glassware in an argon atmosphere in dry, freshly distilled solvents, unless otherwise noted. Yields refer to chromatographically and spectroscopically (^1^H NMR) homogeneous materials unless otherwise stated. Reactions were monitored by thin-layer chromatography (TLC) carried out on 0.15–0.20 mm Yantai silica gel plates (RSGF 254) using UV light (Beijing Tektronix Instrument Co., Ltd., Beijing, China) as the visualizing agent. Chromatography was performed on Qingdao silica gel (160–200 mesh) with petroleum ether (60–90) and ethyl acetate mixtures as eluant. The melting points (mp) were measured on a WRS-1B apparatus (Hangzhou Huier Instrument Equipment Co., Ltd., Hangzhou, China) and were not corrected. ^1^H NMR spectra were obtained with a Bruker AV-300 (300 MHz) (Bruker Corporation, Karlsruhe, Germany). Chemical shifts are recorded in ppm downfield from tetramethylsilane. *J* values are given in Hz. Abbreviations used are s (singlet), d (doublet), t (triplet), q (quartet), b (broad) and m (multiplet). ESI-MS spectra were recorded on a Waters Synapt HDMS spectrometer (Waters Corporation, Milford, MA, USA).

*5,6,7-trihydroxy-2-(4-hydroxyphenyl)-4H-chromen-4-one (2)* Water (10 mL) was added to a stirring mixture of **1** (10.0 g, 21.6 mmol) and concentrated hydrochloric acid (120 mL) in ethanol (120 mL) and the reaction mixture was refluxed under an N_2_ atmosphere for 36 h. After being cooled down to the room temperature, the mixture was poured into water. The solid obtained was filtered, followed by silica gel column chromatographic purification of the residue using 50% ethyl acetate in petroleum ether to afford compound **2** (1.05 g, 17.0% yield) as a yellow solid, mp 160–162 °C. ^1^H NMR (DMSO-*d*_6_, 300 MHz) δ 6.57 (s, 1H, 8-H), 6.74 (s, 1H, 3-H), 6.91 (d, *J* = 8.8 Hz, 2H, 3',5'-H), 7.90 (d, *J* = 8.8 Hz, 2H, 2',6'-H), 8.71 (s, 1H, 6-OH), 10.29 (s, 1H, 7-OH), 10.44 (s, 1H, 4'-OH), 12.78 (s, 1H, 5-OH); ESI-MS: *m*/*z* 287 [M + H]^+^.

*5,6,7-triacetoxy-2-(4-acetoxyphenyl)-4H-chromen-4-one (7)* Pyridine (5 mL) and 4-dimethylaminopyridine (DMAP) (12.2 mg, 0.1 mmol) were added to a stirring solution of **2** (286 mg, 1.0 mmol) in acetic anhydride (5 mL) at 0 °C, then the mixture was warmed to room temperature. After 4 h, the reaction mixture was poured into 100 mL water, and the solid obtained was filtered and purified by silica gel column chromatographic, using 25% ethyl acetate in petroleum ether, to afford compound **7** (408 mg, 90% yield) as a yellow solid, mp 251–252 °C. ^1^H NMR (300 MHz, DMSO-*d*_6_) δ 2.32 (s, 3H, –COCH_3_), 2.35 (s, 3H, –COCH_3_), 2.37 (s, 3H, –COCH_3_), 2.38 (s, 3H, –COCH_3_), 6.95 (s, 1H, 8-H), 7.36 (d, *J* = 8.7 Hz, 2H, 3',5'-H), 7.84 (s, 1H, 3-H), 8.14 (d, *J* = 8.7 Hz, 2H, 2',6'-H); ESI-MS: *m*/*z* 455 [M + H]^+^.

*7-(benzyloxy)-5,6-diacetoxy-2-(4-acetoxyphenyl)-4H-chromen-4-one (8)* K_2_CO_3_ (484 mg, 3.50 mmol, 7.0 equivalent) and benzyl bromide (0.07 mL, 0.60 mmol, 1.2 equivalent) were added to a stirring solution of **7** (227 mg, 0.50 mmol) in dry acetone (20 mL), and then the mixture was refluxed for 6 h and partitioned between 100 mL ethyl acetate and 100 mL water. The ethyl acetate layer was then washed with brine (100 mL), dried over Na_2_SO_4_, filtered, and concentrated. The crude material was purified by column chromatography (25% ethyl acetate in petroleum ether) to yield **8** (186 mg, 74% yield) as a yellow solid, mp 190–191 °C. ^1^H NMR (300 MHz, DMSO-*d*_6_) δ 2.31 (s, 3H, –COCH_3_), 2.32 (s, 3H, –COCH_3_), 2.34 (s, 3H, –COCH_3_), 5.36 (s, 2H, CH_2_), 6.87 (s, 1H, 8-H), 7.36–7.45 (m, 7H, 3',5'-H and ArH), 7.60 (s, 1H, 3-H), 8.13 (d, *J* = 8.8 Hz, 2H, 2',6'-H); ESI-MS: *m*/*z* 503 [M + H]^+^.

*7-(benzyloxy)-5,6-dihydroxy-2-(4-hydroxyphenyl)-4H-chromen-4-one (9)* A solution of sodium hydroxide (0.5 M, 1 mL) was added to a stirring mixture of **8** (100 mg, 0.20 mmol) in methanol (10 mL) at 0 °C under N_2_ atmosphere, after 1h, and the mixture was partitioned between 100 mL ethyl acetate and 100 mL water. The ethyl acetate layer was then washed with brine (100 mL), dried over Na_2_SO_4_, filtered, and concentrated. The crude material was purified by column chromatography (25% ethyl acetate in petroleum ether) to yield **9** (61 mg, 82% yield) as a yellow solid, mp 198–200 °C. ^1^H NMR (300 MHz, DMSO-*d*_6_) δ 5.28 (s, 2H, CH_2_), 6.80 (s, 1H, 8-H), 6.92 (d, *J* = 8.7 Hz, 2H, 3',5'-H), 7.00 (s, 1H, 3-H), 7.35–7.45 (m, 3H, ArH), 7.51–7.54 (m, 2H, ArH), 7.94 (d, *J* = 8.7 Hz, 2H, 2',6'-H), 8.72 (s, 1H, 6-OH), 10.33 (s, 1H, 4'-OH), 12.68 (s, 1H, 5-OH); ESI-MS: *m*/*z* 377 [M + H]^+^.

*7-(benzyloxy)-2-(4-(benzyloxy)phenyl)-5,6-dihydroxy-4H-chromen-4-one (6)* K_2_CO_3_ (109 mg, 0.80 mmol, 1.5 equivalent) and benzyl bromide (0.08 mL, 0.68 mmol, 1.3 equivalent) were added to a stirring solution of **9** (200 mg, 0.53 mmol) in dry DMF (20 mL) at 0 °C, and then the mixture was warmed to room temperature. After 12 h, the reaction mixture was partitioned between 100 mL ethyl acetate and 100 mL water. The ethyl acetate layer was then washed with brine (100 mL), dried over Na_2_SO_4_, filtered, and concentrated. The crude material was purified by column chromatography (25% ethyl acetate in petroleum ether) to yield dibenzylether **6** (210 mg, 85% yield) as a yellow solid, mp 139–141 °C. ^1^H NMR (300 MHz, DMSO-*d*_6_) δ 5.03 (s, 2H, –OCH_2_), 5.23 (s, 2H, –OCH_2_), 6.62 (s, 1H, 3-H), 6.87 (s, 1H, 8-H), 7.18 (d, 2H, *J* = 8.6 Hz, 3',5'-H), 7.31–7.53 (m, 10H, –Ph), 8.04 (d, 2H, *J* = 8.6 Hz, 2',6'-H), 10.82 (s, 1H, 6-OH), 13.11 (s, 1H, 5-OH); ESI-MS: *m*/*z* 465 [M − H]^−^.

*7-(benzyloxy)-2-(4-(benzyloxy)phenyl)-5-hydroxy-6-methoxy-4H-chromen-4-one (10)* K_2_CO_3_ (48 mg, 0.35 mmol, 1.4 equivalent) and iodomethane (0.014 mL, 0.22 mmol, 1.2 equivalent) were added to a stirring solution of **6** (84 mg, 0.18 mmol) in dry DMF (20 mL) at room temperature. After 12 h, the reaction mixture was then partitioned between 100 mL ethyl acetate and 100 mL water. The ethyl acetate layer was then washed with brine (100 mL), dried over Na_2_SO_4_, filtered, and concentrated. The crude material was purified by column chromatography (25% ethyl acetate in petroleum ether) to yield **10** (81 mg, 94% yield) as a yellow solid, mp 196–198 °C. ^1^H NMR (300 MHz, DMSO-*d*_6_) δ 4.00 (s, 3H, –OCH_3_), 4.95 (s, 2H, –OCH_2_), 5.28 (s, 2H, –OCH_2_), 6.62 (s, 1H, 3-H), 6.94 (d, 2H, *J* = 8.6 Hz, 3',5'-H), 7.00 (s, 1H, 8-H), 7.32–7.42 (m, 6H, –Ph), 7.44–7.54 (m, 4H, –Ph), 7.94 (d, 2H, *J* = 8.6 Hz, 2',6'-H), 12.69 (s, 1H, 5-OH); ESI-MS: *m*/*z* 481 [M + H]^+^.

*5,7-dihydroxy-2-(4-hydroxyphenyl)-6-methoxy-4H-chromen-4-one (3)* Ten percent Pd/C (2 mg) was added to a solution of **10** (100 mg, 0.21 mmol) dissolved in ethanol (10 mL) and THF (10 mL) with vigorous stirring. The reaction vessel was then evacuated and the atmosphere replaced with hydrogen. After 8 h, the reaction mixture was filtered and the filtrate was concentrated. The crude material was purified by column chromatography (50% ethyl acetate in petroleum ether) to yield **3** (60 mg, 96%) as a yellow solid, mp 172–174 °C. ^1^H NMR (300 MHz, DMSO-*d*_6_) δ 3.72 (s, 3H, –OCH_3_), 6.58 (s, 1H, 3-H), 6.89 (d, 2H, *J* = 8.6 Hz, 3',5'-H), 7.11 (s, 1H, 8-H), 7.87 (d, 2H, *J* = 8.6 Hz, 2',6'-H), 10.19 (s, 1H, 4'-OH), 10.72 (s, 1H, 7-OH), 12.76 (s, 1H, 5-OH); ESI-MS: *m*/*z* 299 [M − H]^−^.

## 4. Conclusions 

In conclusion, we have efficiently and practically synthesized 6-*O*-methyl-scutellarein (**3**) from scutellarin (**1**) in high yield. Firstly, the acetic anhydride was used to protect all the hydroxyl groups in scutellarein (**2**), and then the benzyl bromide was used to selectively substitute the acetic group at C-7 position in 7. Based on the their reactive activity of the other three hydroxyl groups were different and they were in the preferential order 4' > 6 > 5, benzyl group was used again to protect the C-4' hydroxyl group. This efficient and practical method could be applied to the selective synthesis of other *O*-methylflavonoid isomers, as well as the other flavonoid sulfate- and glucuronide-metabolites *in vivo*. 
